# Differences in motor imagery abilities in active and sedentary individuals: new insights from backward-walking imagination

**DOI:** 10.1007/s00426-023-01876-y

**Published:** 2023-09-29

**Authors:** Laura Mandolesi, Noemi Passarello, Fabio Lucidi

**Affiliations:** 1 Department of Humanities, “Federico II” University of Naples, Via Porta Di Massa, 1, 80133 Naples, Italy; 2 Department of Social and Developmental Psychology, Faculty of Medicine and Psychology, “Sapienza” University of Rome, Via dei Marsi, 78, 00185 Rome, Italy

## Abstract

Evidence has shown that imagining a complex action, like backward-walking, helps improve the execution of the gesture. Despite this, studies in sport psychology have provided heterogeneous results on the use of motor imagery (MI) to improve performance. We aimed to fill this gap by analyzing how sport experience influences backward-walking MI processes in a sample of young women (*n* = 41, mean age = 21 ± 2.2) divided into Active and Sedentary. All participants were allocated to two randomized mental chronometric tasks, in which they had first to imagine and then execute forward-walking (FW) and backward-walking (BW). The Isochrony Efficiency measured the difference between imagination and execution times in both conditions (FW and BW). Moreover, we analyzed the ability to vividly imagine FW and BW within various perspectives in both groups through the Vividness of Movement Imagery Questionnaire (VMIQ-2). Findings showed that active individuals performed better in the BW imagery task when compared to sedentary ones (*F*_1,39_ = 4.98; *p* = 0.03*), while there were no differences between groups in the FW imagery task (*F*_1,39_ = .10; *p* = 0.75). Further, VMIQ-2 had evidenced that the ability to imagine backward is influenced by perspective used. Specifically, the use of internal visual imagery (IVI) led to worse Isochrony Efficiency (*t*_32,25_ = 2.16; *p* = 0.04*), while the use of kinesthetic imagery (KIN) led to better Isochrony Efficiency (*t*_32,25_ =  − 2.34; *p* = 0.03*). These results suggest a close relation between motor experience and complex motor imagery processes and open new insights for studying these mental processes.

## Introduction

Motor imagery (MI) describes the mental execution of a movement, motor act, or action without consciously moving or activating muscles (Decety, 1996; Di Rienzo et al., 2016; Guillot et al., 2008). Findings from neuroimaging and psychophysiological research have shown that both imagination and execution of motor actions engage the same brain networks (Adams et al., 1987; Collet et al., 2011; Decety et al., 1991; Grèzes & Decety, 2001; Guillot et al., 2014; Hanakawa et al., 2008; Hétu et al., 2013; Jeannerod, 1995; Taube et al., 2015).

MI's features enable it to be of value in accelerating procedural learning and recovering motor skills after injuries (Plakoutsis et al., 2022; Ruffino et al., 2017). Thus, MI has been studied extensively in sport psychology since it represents a potentially effective method to promote specific performances in athletes (Fourkas et al., 2008; Louis et al., 2012; Wakefield & Smith, 2012). Among many swimmers, basketball and football athletes often use motor imagery training to perfect their gesture technique (Cuomo et al., 2022). Moreover, it has been observed that by modifying the cerebral activity related to the movement experimentally, MI induces better synchronization of the muscle fibers and inhibition of the antagonist muscle, increasing strength (Morone et al., 2022). Despite all this evidence demonstrating MI training efficacy, research has failed to provide unambiguous experimental protocols, making it difficult to generalize the results (Grealy & Shearer, 2008). Protocols and results heterogeneity can be explained by three main issues: differences in perspective used during MI, differences in athletes’ experience, and the complexity of the gesture imagined (Decety et al., 1989; Kraeutneret al., 2018; Zapala et al., 2021).

MI can be divided into two categories based on the perspective used during imagination: *visual* and *kinesthetic*. Visual imagination of a movement or a sequence of movements can be carried out from a first-person (*internal visual imagery,* IVI) or third-person perspective (*external visual imagery,* EVI) perspective (Yu et al., 2016). In the first-person internal visual perspective, individuals envision themselves from the same viewpoint as the one used during the encoding phase (Nigro & Neisser, 1983; Rice & Rubin, 2009). On the other hand, the third-person visual perspective requires individuals to imagine themselves as onlookers observing the gesture (Weinberg & Gould, 2015). Using this technique, individuals imagine the environment as the “background” of the scene. It has been shown that both IVI and EVI perspectives do not oppose to one another (Rice & Rubin, 2009). Aside from visual modalities, individuals could also use kinesthetic imagery (KIN). KIN imagery involves imagining the feeling of the movement (Roberts et al., 2008). This mechanism improves motor performance solely based on the internal emulation of action (Wilson et al., 2016). Moreover, KIN imagery is likely to involve interoceptive awareness processes (i.e., the ability to perceive internal bodily sensations correctly) (Mehling, 2016), which have already been associated with sport expertise and performance improvement (Kesilmiş & Yıldız, 2018; Ridderinkhof & Brass, 2015; Wallman-Jones et al., 2021). Electrophysiological studies have evidenced differences in brain activity between visual and kinesthetic imagery, observing the effect of manual preference on imagery processes (Zapala et al., 2021).

Athletes’ expertise should also be considered when evaluating the MI process. It has been proven that professional athletes have better imaginative abilities compared to novices since they regularly use MI to improve their gestures (Diotaiuti et al., 2023; Fourkas et al., 2008; Montuori et al., 2018; Wei & Luo, 2010; Zhang et al., 2019). In addition, it seems that experts and novices used different perspectives when imagining (Dana & Gozalzadeh, 2017; Roberts et al., 2008; Williams et al., 2015). Montuori et al. (2018) found that experienced athletes were more efficient at imagining, compared to non-experts, but only when using internal visual imagery. Conversely, non-expert athletes were more efficient when using external visual imagery. In addition, many authors indicated that kinesthetic imagery needs the experience to be effective (Callow & Hardy, 2004; Martini et al., 2016; Robin & Dominique, 2022; Williams et al., 2015).

Lastly, the complexity of the gesture to be imagined represents another crucial issue. Motor behaviors consist of gestures that can be increasingly complex, such as movements, motor acts, and actions (Mandolesi et al., 2018). These motor behaviors may be gained through experience and are added to our *vocabulary of acts* (Rizzolatti et al., 1988). Ideomotor training protocols, like PEETLEP, strongly focus on gesture complexity (Morone et al., 2022). Findings suggest that as the action becomes more complex, more brain systems are involved, resulting in a more extensive activation of large-scale networks (Li et al., 2020). In this context, it is important to underline that action is cognition, that to put into action a motor behavior, it is necessary to plan it (e.g., posterior parietal areas), decide when to execute it (e.g., prefrontal areas), and consider the environmental context (e.g., visual areas). All these mental processes are training with physical exercise. Since MI shares common neural substrates with the preparation and execution of motor action (Jeannerod, 1995; Tomasino & Gremese, 2016), imagination protocols should focus on complex actions to improve gesture speed and accuracy. Performing a sequence of movements *backward* can be a complex action (Wang et al., 2019). Imagining motor behaviors executed backward is difficult and complicated, as it requires a specific cognitive effort regarding working memory, spatial abilities, and executive control (Winter et al., 1989). In this light, there is growing evidence that “backward imagination” could positively affect sport performance and cognition (Aksentijevic et al., 2019; Godde & Voelcker-Rehage, 2017). Godde and Voelcker-Rehage (2017) used fMRI during backward-walking imagination. They found wide activations in the right motor cortex as well as in the superior parietal cortex and precuneus, thalamus, putamen, and caudate nucleus, thus suggesting that imagining action performed backward requires greater cognitive effort than the one forward (Malouin et al., 2010).

The potential benefits of backward MI training and the growing research on this topic, driven, however, by different experimental protocols and methods as well as conflicting findings (Grealy & Shearer, 2008; Guillot & Collet, 2005), led us to conduct a preliminary study to determine if active and sedentary individuals differed in their ability to imagine actions backward. Taking into account the close correspondence between execution and imagination, we hypothesize that people with greater motor experience, such as sportive individuals, are more skilled in complex motor imagination tasks than individuals who do not practice sports.

To this aim, we tested a sample of young women divided into Active and Sedentary in a mental chronometric task in which they had first to imagine and then perform the same motor behavior: forward and backward-walking. Isochrony Efficiency (IE) index was calculated after the task by measuring the difference between imagination and execution times in both conditions (forward and backward).

Moreover, we tested how the ability to vividly imagine a motor behavior within various perspectives (visual internal, visual external, and kinesthetic) differently affected IE for active and sedentary individuals. Given the lack of heterogeneous data on the predominant type of perspective used in MI (Morone et al., 2022), this secondary goal was entirely exploratory. To this aim, we administered the Vividness of Movement Imagery Questionnaire (VMIQ-2) to the same sample. The scores obtained on the three scales of the test—which concerned the use of visual internal imagery, visual external imagery, and kinesthetic imagery during MI (Roberts et al., 2008)—were used as predictors of the imagery task performance within a generalized linear model (GLM). To analyze the influence of sport expertise, we included the interaction between the type of perspective used in MI and group (Active, Sedentary) in our GLM model.

## Materials and methods

### Participants

We recruited 41 women from the “Federico II” University of Naples, divided into two experimental groups: active group (*n* = 20; mean age = 21 ± 1.2); and sedentary group (*n* = 21; mean age = 22 ± 2.3). All participants of the active group had at least 8 continuous years of experience in their sport and trained twice a week (mean years of experience = 6 ± 1.9), whereas sedentary participants had never practiced sport in a structured and constant manner over time.

Inclusion criteria were normal or corrected-to-normal vision and right-handedness. Alternatively, exclusion criteria comprised the current or past presence of psychopathology, psychiatric, neurological, or motor disorders, or other medical illness. The participants were voluntarily enrolled after written informed consent was obtained. The study was approved by the Local Ethics Committee of the “Federico II” University of Naples (protocol number: 11/2020) and carried out in accordance with the Declaration of Helsinki.

### Imagery task

A mental chronometry task was administered to participants to assess their motor imagery abilities. There were two randomly assigned tasks in the procedure: a forward-walking task (FW) and a backward-walking task (BW). In both tasks (each consisting of one trial), participants had first to imagine (forward and backward-walking) and then walk (forwards and backward). The corridor used in the present study was situated within a university building and it was familiar to all participants. According to the procedure used by Grealy and Shearer (2008), participants were not given the opportunity to walk in the corridor immediately preceding the experiment.

At the beginning of the procedure, participants were asked to observe a target (a black leather chair) placed 30 m away from their position. We opted for a distance of 30 m, exceeding that used in other protocols (such as Decety et al., 1989). This choice is rooted in the complexity of imaginative tasks, like the one we employed, which might demand additional time due to the mental manipulations involved in the imagery process. Participants were given the following instructions: “when you are ready, close your eyes and imagine walking (or walking backwards) at your own pace toward the black leather chair”.

For both imagination and execution tasks, each participant was positioned on an “X” placed behind a starting line.

Imagination and execution times were measured using a commercial digital stopwatch (Faviye stopwatch XL 0–13). Participants received instructions for using the stopwatch before the imagination task: they had to close their eyes, put their finger on the start button of the stopwatch, and press it as soon as they started imagining. For the execution phase, time was recorded by the experimenter. The times recorded have been measured in seconds. A few days before the test, participants were informed about MI, its use and benefits, and received minimum information about its execution. Each task was evaluated using an Isochrony Efficiency index (IE = difference between imagination and execution time). The bigger IE index accounted for the worst MI efficiency.

### Self-report assessment

The Vividness of Movement Imagery Questionnaire (VMIQ-2) (Roberts et al., 2008) assessed participants’ abilities to imagine an action vividly. The VMIQ-2 includes 36 items. Participants need to imagine 12 daily actions from three different points of view: external visual perspective (EVI), internal visual perspective (IVI), and kinesthetic perspective (KIN). IVI involves imagining from a first-person perspective, as seeing one's body through one's own eyes, while EVI involves imagining oneself from a third-person perspective, like watching oneself on a video. Lastly, KIN imagery involves imagining the feeling of the movement, as if the body is moving during imagination. Participants have to rate the vividness of the imagined action on a Likert scale from 1 (perfectly clear and vivid) to 5 (no imagination—I only know that I am thinking about the action). Items are the following: daily actions (walking, running); actions that involve precision (kicking a stone, bending to pick up a coin); actions that involve overcoming an obstacle (running upstairs, jumping sideways); actions that involve manipulation of objects (throwing a stone into water, kicking a ball in the air); fast actions that involve balance (running downhill, riding a bike), and actions that involve the control of objects/balance in the air (swinging a rope, jumping off a high wall). In this study, we used the Italian version of the VMIQ-2. Test reliability was assessed using the split-half procedure (*r* = 0.76) (Di Corrado et al., 2019).

### Data analysis

One-way ANOVA was used to separately analyzed differences between groups (Active, Sedentary) in the forward and the backward imagery task. *Post-hoc* analyses were conducted through Tukey tests. 95% confidence intervals lower and upper bounds were also considered. The effect size for each ANOVA was calculated through *ω*^2^. Shapiro–Wilk test was used to assess data normality (IE forward: *p* = 0.17; IE backward: *p* = 0.35), while Levene test was used to assess quality of variance (IE forward: *p* = 0.72; IE backward: *p* = 0.22).

To investigate whether imagination perspectives (internal, external, kinesthetic) affect the performance of active and sedentary participants differently, we performed a generalized linear model (GLM) (Dunn & Smyth, 2018) using scores of the VMIQ-2 scales (IVI, EVI, KIN) as continuous predictors, group (Active, Sedentary) as the categorial predictor, and forward and backward IE indexes as the dependent variable. 95% confidence intervals lower and upper bounds were also considered.

All analyses and graph illustrations were conducted through JASP software 0.17.2.1.

Due to our relatively small sample size, we conducted a post-hoc power analysis, which revealed a power level (1- β) of 0.34.

## Results

**Fig. 1:**
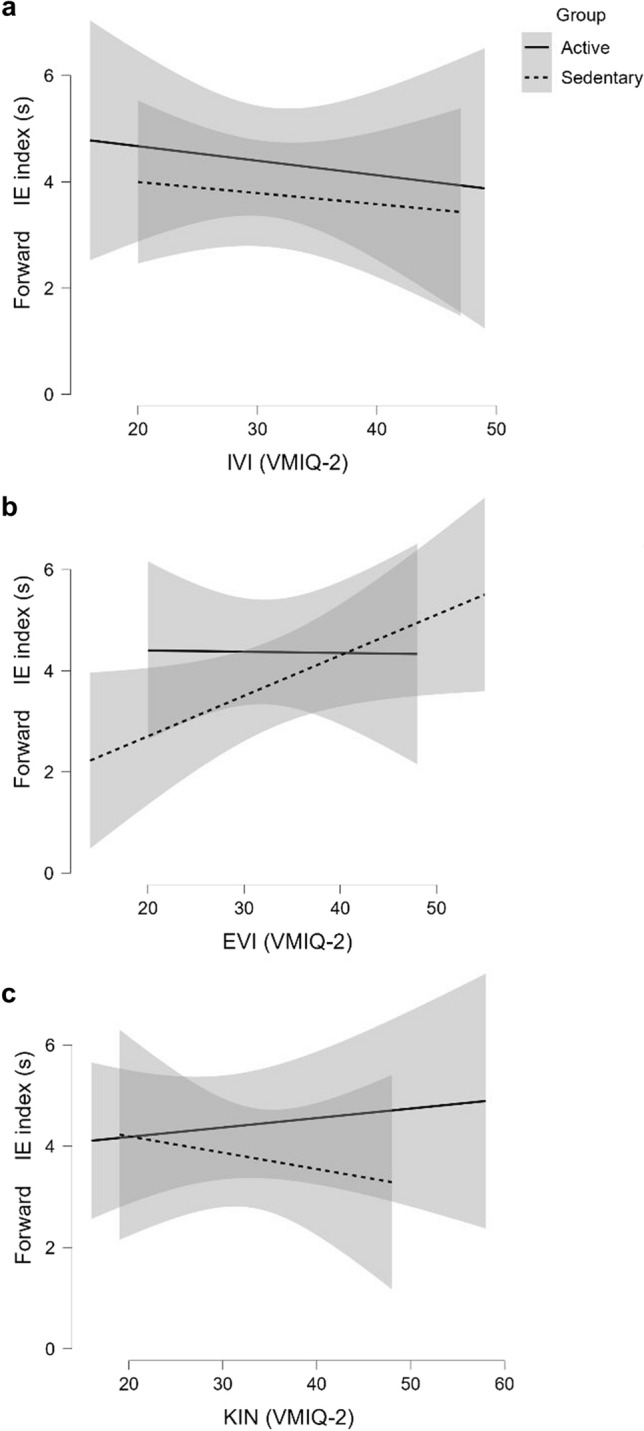
One-way ANOVA results. **a** results on Forward MIE index (motor imagery index); **b** results on the Backward IE index

One-way ANOVA outputs showed no significant effect for the group on the forward IE index (F_1,39_ = 0.10; *p* = 0.75; *ω*^2^ = 0.01) (Fig. 1a). Conversely, a significant effect for the group was found on the backward IE index (*F*_1,39_ = 4.99; *p* = 0.03*; *ω*^2^ = 0.09). *Post-hoc* analysis through the Tukey test revealed that the Sedentary exhibited less motor imagery efficiency (bigger IE = larger difference between imagination and execution time) than the Active (mean difference = 2.96; LCI = 0.28; UCI = 5.64; *t*_1,39_ = 2.23; *p*_*tukey*_ = 0.03*) (Fig. 1b). All results are reported in Table 1.

**Table 1:** One-way ANOVA (Active, Sedentary) results on motor imagery task. *Post-hoc* analyses with the Tukey test and 95% confidence interval lower (LCI) and upper (UCI) bound are reported

	*F*_1,39_	*p*	*p*_Tukey_	*ω*^2^	95% LCI	95% UCI
IE^a^ forward	0.10	0.75	0.75	0.01	− 1.20	1.65
IE^a^ backward	4.98	0.03*	0.03*	0.09	0.28	5.64

IE^a^ Isochrony Efficiency index

*p < 0.05

**Fig. 2:**
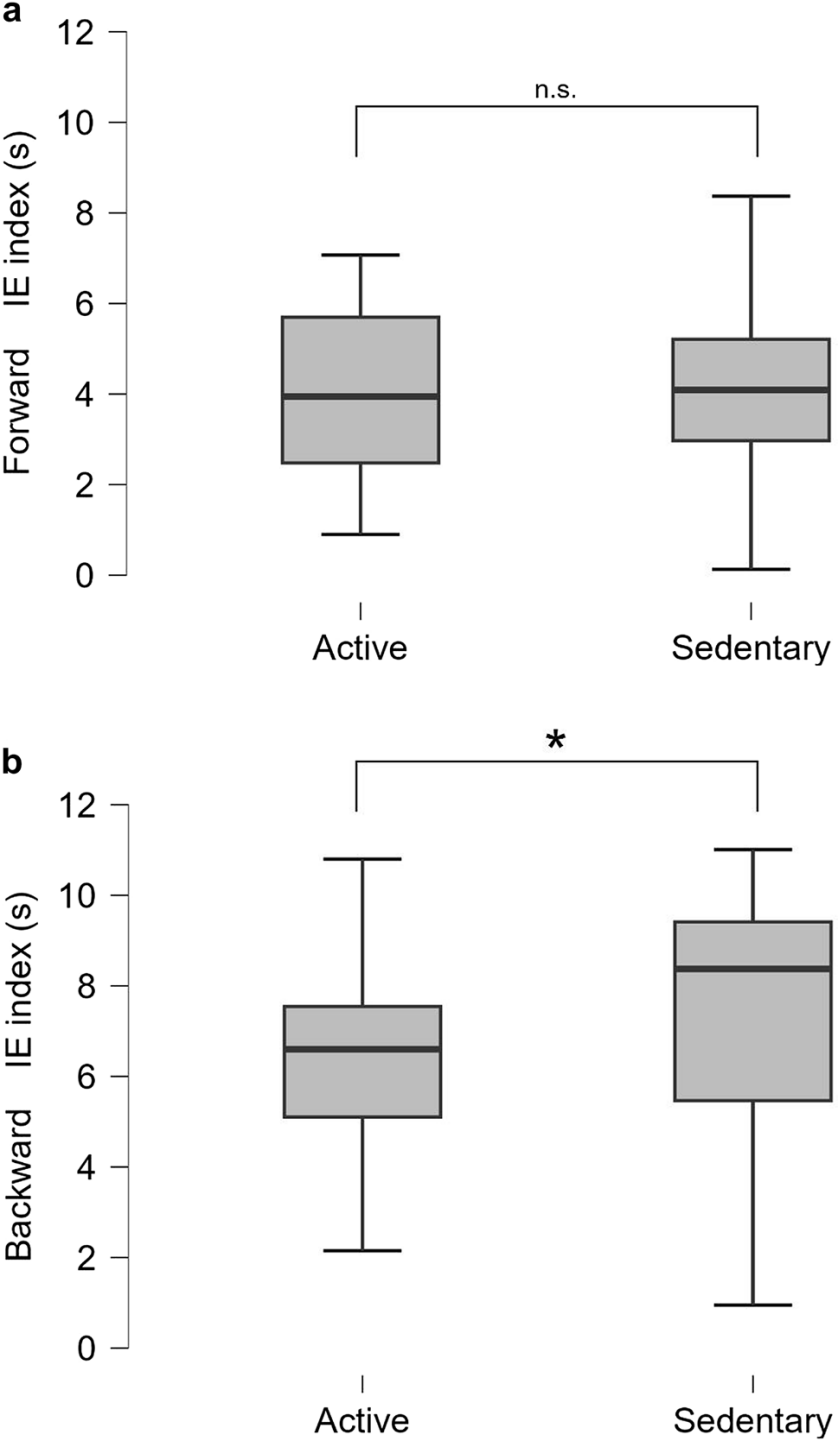
GLM results on Forward IE index with IVI (**a**), EVI (**b**), and KIN (**c**) as predictors

**Fig. 3:**
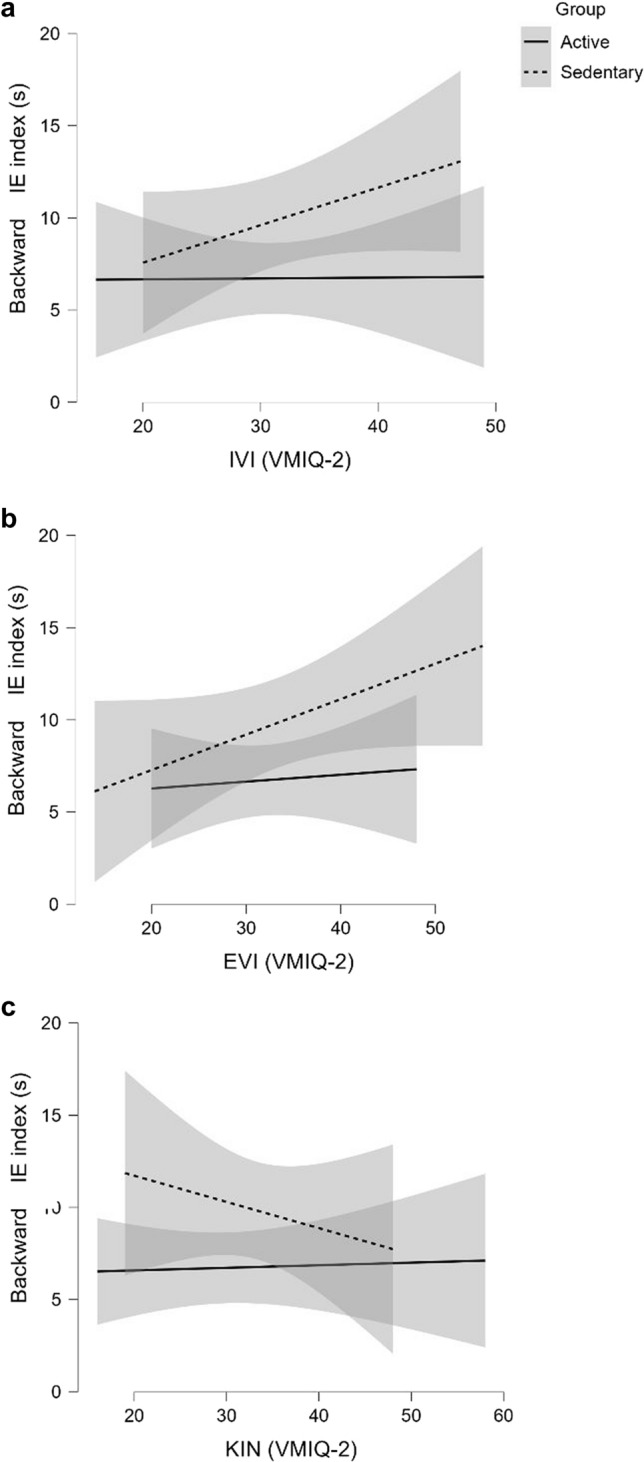
GLM results on Backward IE index with IVI (**a**), EVI (**b**), and KIN (**c**) as predictors

According to our GLM model, the ability to vividly imagine actions from an external perspective (EVI) predicted the IE index in the forward task (*t*_32,25_ = 2.44; *p* = 0.02*; LCI = 0.02; UCI = 0.21) (Fig. 2b). No significant effect for group and group -VMIQ-2 interaction was found. Conversely, the ability to vividly actions from an internal (IVI) and kinesthetic (KIN) perspective predicted IE index in the backward task (IVI: *t*_32,25_ = 2.16; *p* = 0.04*; LCI = 0.03; UCI = 0.61; KIN: *t*_32,25_ = -2.34; *p* = 0.03*; LCI =  − 0.67; UCI =  − 0.06) (Fig. 3a, c). Significant effect was found for group, and KIN interaction (*t*_32,25_ = 2.04; *p* = 0.05*; LCI = 0.02; UCI = 0.75) (Fig. 3c). All GLM outputs are shown in Tables 2 and 3.

**Table 2:** GLM results for Forward IE index

Predictors	*t*_32,25_	*p*	Estimate	95% LCI	95% UCI
IVI	− 1.41	0.17	− 0.09	− 0.23	0.04
EVI	2.44	0.02*	0.12	0.02	0.21
KIN	0.18	0.86	0.01	− 0.13	0.15
IVI x Group	0.12	0.90	0.01	− 0.19	0.21
EVI x Group	− 1.73	0.09	− 0.12	− 0.26	0.02
KIN x Group	0.46	0.65	0.04	− 0.13	0.21

Estimate, *p*- significance, and 95% confidence interval lower (LCI) and upper (UCI) bound are reported

*IVI* internal visual imagery, *EVI* external visual imagery, *KIN* kinesthetic imagery

*p < 0.05

**Table 3:** GLM results for Backward IE index

Predictors	*t*_32,25_	*p*	Estimate	95% LCI	95% UCI
IVI	2.16	0.04*	0.32	0.03	0.61
EVI	0.79	0.43	0.08	− 0.12	0.29
KIN	− 2.34	0.03*	− 0.37	− 0.67	− 0.06
IVI x Group	− 1.53	0.14	− 0.34	− 0.78	0.09
EVI x Group	− 0.31	0.75	− 0.05	− 0.35	0.26
KIN x Group	2.04	0.05*	0.38	0.02	0.75

Estimate, p- significance, and 95% confidence interval lower (LCI) and upper (UCI) bound are reported

*IVI* internal visual imagery, *EVI* external visual imagery, *KIN* kinesthetic imagery

*p < 0.05

## Discussion

The present study aimed to gain insight into complex action imagery processes, such as backward-walking, as they can be useful in developing training protocols based on MI. Specifically, our main hypothesis concerns the influence of physical activity and sport performance on MI abilities. We tested a sample of young women, divided into Active and Sedentary, in a mental chronometric task in which they had first to imagine and then execute a simple (forward-walking) and a complex (backward-walking) motor behavior. Isochrony Efficiency index (IE), calculated as the difference between imagination and execution times in both conditions (forward and backward), was used.

Our findings showed that active individuals, who are experienced in sports, performed better in backward-walking imagination than Sedentary. Active individuals showed a superior IE index (i.e., a smaller difference between imagination and execution times) (Table 1; Fig. 1b). Interestingly, no significant differences were found between groups in the forward task (Table 1; Fig. 1a).

These results can be discussed in relation to the physical and mental features of both tasks (forward and backward), considering that task difficulty influences temporal accuracy (Calmels & Fournier, 2001). Forward-walking (FW) is a simple, innate, and automatized motor behavior peculiar to humans (Aksentijevic et al., 2019). As such, FW—and therefore FW imagination—is a skill mastered by all individuals, even sedentary ones. On the other hand, backward-walking (BW) is not viewed as a simple reversal of forward motion, but it can be considered a complex motor behavior (Suenaga et al., 2013) that requires specific abilities, such as cognitive, sensory, perceptive, and interoceptive abilities. During BW, individuals need to rely on other sensorial systems, aside from the visual one, since they do not have a complete view of the road and the obstacles ahead. It is a complex construct incorporating multiple biomechanical, neurological, and sensory systems (Winter et al., 1989). As previously mentioned, imagination and execution share the same neural circuits (Jeannerod, 1995). Therefore, imagining an action performed backward could activate large-scale brain networks (Godde & Voelcker-Rehage, 2017). Backward imagery requires not only planning, scheduling, decision-making, and choosing the best body schema to use but also working memory, spatial processing, and procedural memory. Aksentijevic and colleagues (2019) referred to backward imagery as the “Mnemonic Time Travel Effect” since it recalls action timing processes associated with gesture memory and the analysis of the environment in which the individual is located while imagining. To underline the effectiveness of complex backward motor behaviors on sport performance, we can consider a recently developed sport specialty, backward running. Several studies have shown that this discipline is very effective because positively affects mental and physical abilities (Suenaga et al., 2013; Wang et al., 2019). Thus, investigating complex MI, such as backward imagination, emerges as a key factor in improving physical and mental training in athletes to achieve success in sport.

The better performance of active individuals in comparison to Sedentary in the backward imagination task can be explained by the fact that physically more active people (like our Active group) physically train different fine motor skills more often, so they have a better, more detailed, and more varied representation of different fine motor skills. The present finding is in line with previous results that evidenced how motor experience is an essential factor for the accuracy of imagery timing (Calmels & Fournier, 2001; Grealy & Shearer, 2008; Ladda et al., 2021; Montuori et al., 2018). In this context, studies have also evidenced that athlete experts have an accurate temporal congruence in comparison to novices (Guillot & Collet, 2005). It could also be read in neurobiological terms, namely brain activity, especially in motor areas, is typically greater in athletes experts than in non-experts during motor imagery tasks (Mizuguchi & Kanosue, 2017), suggesting thus that sport favors an enhancement of MI, as well as, in the same way, MI train motor abilities.

Our secondary hypothesis regarded the ability to vividly imagine motor behaviors within various perspectives (visual internal, visual external, and kinesthetic) in sedentary and active individuals and how the use of different perspectives was influenced by physical activity and sport experience. We administered the Vividness of Movement Imagery Questionnaire (VMIQ-2) to active and sedentary participants and used VMIQ-2 scales as predictors for MIE in a generalized linear model (GLM).

GLM outputs (Table 2) on the forward task showed no association between the IE index and both visual internal (IVI) and kinesthetic (KIN) perspectives. However, the visual external (EVI) perspective predicted active and sedentary MI abilities. Specifically, the use of the EVI perspective led to worse MI efficiency (bigger IE index) (Fig. 2b). Further, in backward task, both IVI and KIN perspectives predicted active and sedentary performance (Table 3). IVI perspective led to worse MI efficiency (bigger IE index) (Fig. 3a), while the use of KIN led to better MI efficiency (smaller MEI index). KIN influence on MI efficiency was true for Sedentary but not for Active (Fig. 3c).

These findings showed that the ability to imagine is indeed influenced by the perspective used. Nevertheless, this seems to apply mostly to sedentary individuals rather than active ones. The association between EVI perspective and poor imagery abilities in FW may be explained by the fact that FW is a simple motor pattern. Therefore, reproducing it from the perspective typically used for learning new movements was inefficient (Montuori et al., 2018). However, results on BW appear to confirm the evidence that the KIN is more useful for improving MI abilities. Contrary to previous findings (Dahm, 2022), KIN did not primarily affect active individuals’ performance. Studies have shown that athletes use KIN mostly to improve the execution of specific gestures. Even if comparing self-report and easy mental chronometry tasks of EVI, IVI, and KIN perspectives, Williams and collaborators (2015) found that elite athletes had significantly higher KIN scores than IVI and EVI for self-report measures. As our sample was made up of sportives playing heterogeneous sports—and not all were practicing BW—so we can speculate that the impact of kinesthetic imagination was not prominent. Further, Fusco and colleagues (2014) have found that athletes’ temporal congruence in BW was improved only when using *dynamic motor imagery* (dMI). As opposed to static MI, in dMI, athletes can execute small movements (i.e., steps) while imagining walking backward. Lastly, Morone and colleagues (2022) argued that it might be beneficial for athletes to combine alternative perspectives together instead of using preferentially only one. In future, homogeneous athletes' samples should be used to confirm or disprove this finding.

The use of MI in sport psychology remains of particular interest, despite all methodological issues discussed so far. MI could be essential for creating training protocols suitable for beginners or more experienced athletes (Fusco et al., 2014; Montuori et al., 2018; Dello Iacono et al., 2021) and for developing rehabilitation therapies for elderlies or individuals with psychomotor disorders (Passarello et al., 2022a, 2022b). As stated by Morone and colleagues (2022), MI practice could help with motor skills acquisition by enhancing brain plasticity processes that have been long associated with sport practice and physical exercise (Mandolesi et al., 2018; Passarello et al., 2022a, 2022b). Moreover, the effects of MI on brain plasticity have been highlighted by studies on MI-based rehabilitation practice of subjects losing motor skills in their limbs (Burianová et al., 2016, 2020). In this context, Ruffino and colleagues (2019) argued that MI could intervene in cortical reorganization processes associated with behavioral improvement. Recently, several studies have supported the potential beneficial effects of motor imagery interventions in clinical populations with locomotion deficits such as Parkinson’s disease (Cuomo et al., 2022).

### Limitations and future directions

Given its preliminary nature, this study presents some limitations. First, our sample consisted entirely of young adult women. Since gender differences significantly influence the processes of mental imagery, testing young adult men is essential to gain stronger insights and extend these results to the general population. Another limit concerns the sample size and the type of action imagined backward. We have chosen a complex imagining task, such as walking backward. Since the distance to be covered appears to be an essential factor in the equivalence of motor imagery timing (Papaxanthis et al., 2002), it might be useful for the next studies to imagine this protocol with a greater distance. Similarly, even the perceived effort modulates MI (Grealy & Shearer, 2008), and therefore, inserting a variable such as weight in our tasks, according to the Dacety et al. (1989) protocol, would help better to provide interesting insights into the nature of motor imagery timing. In future, it would be useful to analyze different athletes of different disciplines (open-skill and closed-skill sports) and calculate the imagery timing estimates to evaluate overestimation and underestimation in relation to the motor experience. Also, future studies should evaluate different types of backward actions, even of increasing difficulty. Finally, it might be useful to use a protocol that includes more trials to observe the effects of the practice on backward imagery processes.

## Conclusion

The action is cognition, and sport practice enhances the interaction of multiple cognitive domains that allow the action to be accurate, effective, and fast. As physical activity improves both cognitive and motor functions, people who engage in sports will perform better and mentally represent complex actions, such as walking backward. There is a lot of evidence in sport psychology that documents how individuals who participate in sport activities have better cognitive abilities than sedentary ones, regardless of age (Mandolesi et al., 2018; Passarello et al., 2022a, 2022b; Serra et al., 2021). Although we have not evaluated the singular cognitive domains required for a complex mental representation task, we believe this is the reason why our group of active individuals was favored in the backward imaginative task in comparison to sedentary ones. Surely experience and sports practice are other factors that explain why active individuals performed better in the backward imagination task. Indeed, the more active people physically train in increasingly complex sequences of movements. This fact leads to a better mental representation of the different motor schemes.

Moreover, we evidenced that our ability to imagine generally depends on the perspective used for imagining, suggesting, thus, specific imagery protocols related to experience in sport. Although preliminary, our study provides a contribution to the development of motor imagery protocols based on backward action that could improve efficiency in sports and other fields of application of psychology and neurorehabilitation.

## Data Availability

The data are available from the corresponding author upon request**.**
